# Determinants of Depression in Caregivers of Geriatric Patients in Jeddah, Saudi Arabia: A Cross-Sectional Study

**DOI:** 10.3390/medicina60081368

**Published:** 2024-08-22

**Authors:** Mohammed A. Aljunaid, Rayan Mesfer Alosaimi, Essa Ahmed Alazmi, Ahmad Abdulaziz Afandi, Mohammed Talal Musslem, Mohammed Mohsen Aljarameez, Hosam Husain Alzobaidi

**Affiliations:** 1 Department of Family and Community Medicine, University of Jeddah, Jeddah 21589, Saudi Arabia; 2 Faculty of Medicine, University of Jeddah, Jeddah 21589, Saudi Arabiamohammedaljarameez@gmail.com (M.M.A.);

**Keywords:** caregivers, geriatric patients, depression, mental health, Zarit burden interview

## Abstract

*Background and Objectives*: Caregiving for geriatric patients is essential for ensuring the well-being and quality of life of older adults. Family caregivers play a crucial role, but they often face a significant burden that can lead to adverse mental health outcomes, including depression. This study aimed to estimate the prevalence of depression among caregivers of geriatric patients in Jeddah, Saudi Arabia, and to analyze its association with caregiver burden and various socio-demographic and caregiving parameters. *Methods*: A cross-sectional study was conducted in Jeddah, Saudi Arabia, between January and March 2024. Adult caregivers of geriatric patients were recruited through various social media platforms. Data were collected via an electronic questionnaire that included demographic information, caregiving parameters, the Patient Health Questionnaire-9 (PHQ-9) for depression screening, and the Zarit Burden Interview (ZBI-12) for caregiver burden assessment. Data were analyzed using descriptive statistics, chi-square tests, and multivariate logistic regression. *Results*: Of the 269 participants, the average age was 32 years, and the gender distribution was nearly balanced. The prevalence of depression (PHQ-9 score ≥ 10) among caregivers was 45.4% (95% CI: 39.3, 51.5%). Significant factors associated with higher depression scores included younger age, female gender, single status, being a student, low income, and caregiving burden. In the multivariate analysis, female gender (OR 2.50, 95% CI 1.30–4.80) and caregiving burden (mild-to-moderate burden: OR 6.18, 95% CI 2.94–13.00; high burden: OR 22.75, 95% CI 8.75–59.13) were independent predictors of depression. *Conclusions*: The study highlights the high prevalence of depression among caregivers of geriatric patients in Jeddah and underscores the significant impact of caregiving burden on mental health. These findings emphasize the need for targeted interventions, such as mental health support, respite care programs, and culturally sensitive educational training, to mitigate caregiver burden and enhance the well-being of caregivers.

## 1. Introduction

Caregiving in the context of geriatric patients is essential for ensuring the well-being and quality of life of older adults. It involves providing not only physical assistance but also emotional support, companionship, and advocacy for the needs of older individuals. Family caregivers, in particular, play a crucial role in supporting geriatric patients, especially as the population ages and the demand for long-term care increases [1]. Furthermore, caregivers often act as advocates for older adults, ensuring their preferences are respected in healthcare decision-making [2].

Caregiver burden encompasses the physical, emotional, social, and financial stressors faced by individuals providing care to impaired or ill individuals, resulting in an imbalance between caregiving demands and personal resources [3]. Juggling caregiving responsibilities with personal and professional commitments can be overwhelming, leading to caregiver burden and fatigue [4]. The negative reactions experienced by caregivers due to the demands of caregiving can lead to burnout and other adverse consequences, potentially affecting their ability to provide care [5,6,7]. Hence, this burden significantly influences the potential need for patient institutionalization [8], which may have a snowballing effect on healthcare system efficiency and the economy.

The relevance of studying caregiving burden in the context of geriatric patients arises from the increasing age-related morbidity and its associated health and economic burdens on societies. In Saudi Arabia, the aging population is projected to lead to a significant rise in the incidence of first strokes in the coming decade [9]. Moreover, despite only 3.2% of the Saudi population being over 65 years old, the prevalence of chronic conditions poses a considerable risk of severe complications in older individuals. Metabolic diseases, cancer, and chronic obstructive pulmonary disease (COPD) are on the rise, indicating a growing disease burden associated with aging [10,11,12]. Additionally, cardiovascular diseases, myocarditis, and thyroid cancer show age-specific patterns, with age-standardized rates reflecting the impact of aging on these conditions [13,14,15]. These figures are compounded by the increasing life expectancy, a key component of Saudi Vision 2030 [16]. Maintaining longevity and healthiness in line with this vision requires considering macroeconomics, socio-demographics, and health resources in shaping a population’s health and behavior [17].

While several studies have explored mental illness among caregivers of elderly individuals in Saudi Arabia, data regarding the relationship between such disorders and caregiving burden are scarce [18]. Therefore, we conducted this study to estimate the prevalence of depression among caregivers of geriatric patients in Jeddah, Saudi Arabia, and to analyze its association with caregiver burden and various socio-demographic and caregiving parameters. Understanding the extent and impact of caregiver burden, particularly its correlation with depression, is crucial for developing targeted interventions that can mitigate these challenges. While anxiety and burnout are also important concerns and should be explored in other studies, depression was chosen as the focus of this study because it is a particularly common and impactful mental health issue that significantly affects caregivers’ well-being. Such data will provide valuable insights into the specific needs and stressors faced by caregivers, enabling healthcare providers and policymakers to devise effective support systems. Furthermore, this research can inform strategies to enhance the well-being of both caregivers and geriatric patients, ultimately improving the overall efficiency of the healthcare system and reducing economic burdens.

## 2. Methods

### 2.1. Design and Settings

A cross-sectional study was conducted among the population of Jeddah, Saudi Arabia, between January and March 2024. The study was ethically approved by the institutional review board of the University of Jeddah (#UJ-REC-147).

### 2.2. Participants

The study targeted adult (18 years or older) residents of Jeddah who provide caregiving for a geriatric relative or patient. The study excluded individuals with conditions that impede communication or compromise the reliability of their responses, such as cognitive impairments or severe mental health conditions. Additionally, individuals who refused to participate were also excluded.

### 2.3. Sampling

Participants were recruited via various social media platforms. A convenience sampling technique was first employed to include all consenting participants. The recruitment strategy was complemented with a snowball sampling technique, where initial participants were encouraged to share the questionnaire with their own networks. 

### 2.4. Data Collection

The data collection tool used in this study was an electronic questionnaire designed to gather information about the participants and their caregiving experiences. It comprised 4 parts:

- Part 1 collected demographic data such as age, gender, marital status, education level, professional status, and monthly income.
- Part 2 focused on information on the relationship of the caregiver to the patient, caregiving frequency, living arrangements, caregiving mode, financial responsibility, and duration of caregiving.
- Part 3 consisted of a slightly modified version of the Patient Health Questionnaire-9 (PHQ-9) scale used to screen for depression. The modification involved a cultural adaptation of the 9th item, which was rephrased from “Thought that you would be better off dead, or of hurting yourself” to “Feeling as though life isn’t worth living”. The Arabic version was shown to be reliable and valid in the Saudi population [19]. The PHQ-9 questionnaire is a widely used scale with validated use across various populations. A meta-analysis including 58 eligible studies with a total of 17,357 participants and 2312 cases of major depression evaluated the diagnostic accuracy of the PHQ-9 for detecting major depression. This study found that a cut-off score of 10 or above maximized the combined sensitivity (0.88, 95% CI 0.83 to 0.92) and specificity (0.85, 95% CI 0.82 to 0.88) among studies using semi-structured diagnostic interviews. Sensitivity was higher with semi-structured interviews compared to fully structured interviews and the Mini International Neuropsychiatric Interview (MINI). The PHQ-9 demonstrated similar sensitivity but potentially lower specificity for younger patients compared to older patients. Therefore, a cut-off score of 10 or above is recommended for use across different age groups and diagnostic settings [20]. The cut-off of 10 or above is relatively contested, with some studies showing that the cut-off is between 8 and 11. Therefore, the use of PHQ-9 should be complemented with a clinical assessment by a mental health professional [21].
- Part 4 assessed the caregiving burden using the short form of the Zarit Burden Interview (ZBI-12), which comprises 12 items. Each item is rated on a scale from “Never” to “Almost always”, reflecting how frequently caregivers experience specific challenges, such as lack of personal time, anger near the relative, health impact, etc. An overall ZBI-12 score is computed and interpreted as follows: none-to-mild burden (0–10), mild-to-moderate (>10–20), and high (>20). This scale has excellent psychometric properties [22] and has been validated for assessing caregiver burden across various populations and caregiving settings [23,24].

### 2.5. Procedure

The questionnaire was edited for electronic administration using Google Forms. The link was distributed via social media platforms, including Facebook, Twitter, and LinkedIn, employing a snowball sampling technique to broaden reach. Participation was voluntary, with electronic informed consent obtained at the beginning. Confidentiality and anonymity were assured, with no personal identifying information collected.

### 2.6. Variables and Outcome

The dependent variable, being the primary outcome, is the depression status assessed using the PHQ-9 score and defined as a score ≥ 10. The other variables, including the caregiver’s demographic factors, caregiving parameters, and the ZBI-12 score, were analyzed as the independent variables.

### 2.7. Statistical Methods

Data analysis was performed using IBM SPSS Statistics for Windows, Version 21.0 (IBM Corp., Armonk, NY, USA). Descriptive statistics were used to summarize the demographic characteristics, caregiving parameters, and responses to the PHQ-9 and the ZBI-12 scales. Means and standard deviations were calculated for continuous variables, while frequencies and percentages were used for categorical variables. The independent t-test was utilized to compare mean ages between groups with different depression statuses. Chi-square tests were employed to examine the associations between categorical variables and depression status, defined by a PHQ-9 score of 10 or higher. Multivariate logistic regression, using the Enter method, was conducted to identify independent predictors of depression among caregivers, with results presented as odds ratios (ORs) and 95% confidence intervals (CIs). Variables included in the regression model were selected based on their significance in univariate analyses and theoretical relevance. The multivariate model’s explanatory power was assessed using the coefficient of determination (R²). Statistical significance was set at *p* < 0.05 for all tests.

## 3. Results

### 3.1. Participant Characteristics

Of 273 responses received, 4 (1.5%) did not provide consent for participation. This resulted in a total of 269 participants. The average age of the participants was approximately 32 years (SD = 15.01), ranging from 12 to 77 years. Gender distribution was nearly balanced. A majority were single (63.6%), followed by married (29.7%). Regarding education, the largest groups had completed a bachelor’s degree (38.7%) or were college students (30.5%). Regarding professional status, 42.0% were students, and 30.1% were employed. Monthly income data showed that 28.3% of the participants earned more than 10K SAR, while 33.1% earned less than 3K SAR (Table 1).

### 3.2. Caregiving Patterns

Among caregivers, 49.4% were sons or daughters of the patients, and the majority (65.1%) had been providing care for more than six months. The majority (60.6%) provided care daily, with 21.9% doing so for more than 8 h daily, and 58.7% lived with the patient. The role of sole caregiver was less common, with 81.4% sharing caregiving duties. Regarding financial responsibility, only 25.7% declared being financially responsible for the patient (Table 2).

### 3.3. Depression Assessment Using PHQ-9 Scale

The patterns of answers to the PHQ-9 items are depicted in Table 3. Key findings included frequent feelings of tiredness or low energy, with a mean score of 2.36, and difficulty sleeping, with a mean score of 2.26. Losing interest in activities (mean score of 2.09) and trouble concentrating (mean score of 2.02) were also common. The least frequent symptoms were feeling life is not worth living (mean score of 1.66) and moving or speaking slowly or restlessly (mean score of 1.73). The PHQ-9 showed high reliability with a Cronbach’s alpha of 0.890.

The assessment of total PHQ-9 scores showed a mean of 8.93 (SD 6.53) and a median of 9 (range 0–27). Normality testing indicated that the data were not normally distributed (Kolmogorov–Smirnov 0.099, *p* < 0.001; Shapiro–Wilk 0.955, *p* < 0.001). Using a cut-off score of ≥10, the prevalence of depression was found to be 45.4% (95% CI: 39.3, 51.5%).

### 3.4. Assessment of Caregiving Burden

In the assessment of caregiving burden using the ZBI-12 scale, the most concerning items were the feeling of needing to do more for the relative (23.0% often or almost always, mean score 2.49) and feeling they could have taken better care of the relative (25.6% often or almost always, mean score 2.49). Conversely, the least concerning items were the impact on caregivers’ health (6.0% often or almost always, mean score of 1.65) and feeling angry near their relative (7.0% often or almost always, mean score of 1.76). The scale demonstrated high reliability with a Cronbach’s alpha of 0.912. For detailed item responses, see Table 4.

The assessment of overall ZBI-12 scores revealed the following distribution among participants: 152 individuals (56.5%) experienced none-to-mild burden (scores 0–10), 61 individuals (22.7%) reported mild-to-moderate burden (scores > 10–20), and 56 individuals (20.8%) experienced high burden (scores > 20).

### 3.5. Factors Associated with Likelihood of Depression among Caregivers

Factors associated with a higher likelihood of depression (PHQ-9 score ≥ 10) are presented in Table 5. Younger age was significantly associated with higher depression scores (mean age 27.84 vs. 35.37 years, *p* < 0.001). Females were more likely to have depression compared to males (56.2% vs. 43.8%, *p* < 0.001). Marital status was also significant, with single individuals showing a higher likelihood of depression (55.6%, *p* < 0.001) compared to their counterparts. Professional status indicated that students had a higher depression prevalence (59.3%, *p* = 0.001). Income levels revealed that those earning less than 6K SAR had higher depression rates (56.2% for <3K SAR and 56.9% for 3K-6K SAR, *p* = 0.001). Regarding caregiving modalities, providing care for less than 8 h daily was associated with the highest likelihood of depression (53.8%), compared to more than 8 h daily (32.2%) or not providing daily care (44.3%) (*p* = 0.028). The likelihood of depression was proportional to the level of caregiving burden, with caregivers experiencing none to mild burden (Zarit-12 score 0–10) showing a 25.0% likelihood of depression, while those with high burden (Zarit-12 score > 20) showed an 83.9% likelihood of depression (*p* < 0.001).

The bivariate correlation analysis of PHQ-9 and Zarit-12 scores showed a moderate positive correlation with Spearman’s rho = 0.544 (*p* < 0.001).

### 3.6. Independent Factors of Depression

Female gender was independently associated with a higher likelihood of depression (OR 2.50, 95% CI 1.30–4.80, *p* = 0.006). Caregiving frequency was also significant, with caregivers providing care for less than 8 h daily (OR 4.21, 95% CI 1.69–10.49, *p* = 0.002) and those not providing daily care (OR 3.76, 95% CI 1.51–9.35, *p* = 0.004) being more likely to experience depression compared to those providing more than 8 h of daily care. Caregiving burden was also a strong predictor of depression. Those with mild-to-moderate burden had an OR of 6.18 (95% CI 2.94–13.00, *p* < 0.001), and those with high burden had an OR of 22.75 (95% CI 8.75–59.13, *p* < 0.001), compared to those with none-to-mild burden. Other factors such as age, marital status, professional status, and monthly income did not show significant associations in this model. The model explained 46.4% of the variance in the likelihood of depression (R² = 0.464). For detailed statistical values and further associations, refer to Table 6.

## 4. Discussion

### 4.1. Summary of Findings

This study aimed to explore the prevalence and factors associated with depression among caregivers of geriatric patients in Jeddah, Saudi Arabia, highlighting the relevance of understanding caregiver burden and its impact on mental health. The study involved 269 participants, with an average age of 32 years, a balanced gender distribution, and the majority being single (63.6%). Caregivers were primarily sons or daughters (49.4%) of the patients, with 65.1% providing care for over six months. Most caregivers (60.6%) provided daily care, and 58.7% lived with the patient. Shared caregiving duties were common (81.4%), and only 25.7% had financial responsibility for the patient. Based on the PHQ-9 scale, the most frequent depressive symptoms were tiredness or low energy and difficulty sleeping. The overall prevalence of depression (PHQ-9 score ≥ 10) was 45.4% (95% CI: 39.3, 51.5%). Caregiving burden assessed using the ZBI-12 scale indicated that 20.8% of caregivers experienced high burden, while 22.7% reported mild-to-moderate burden. The most concerning items were the feelings of needing to do more and having taken better care of the relative. While several factors were associated with higher depression likelihood, the multivariate model identified female gender, caregiving frequency, and burden as independent predictors, explaining 46.4% of the variance in depression likelihood. Notably, caregiving frequency showed a paradoxical finding where caregivers providing care for less than 8 h daily or not providing daily care had a higher likelihood of depression compared to those providing care for more than 8 h daily.

### 4.2. The Burden of Care: Depression and Caregiving

The prevalence of depression among caregivers of geriatric patients is a significant concern that has been extensively studied in various contexts. Studies have consistently shown that caregiving can lead to depression among caregivers, with some research indicating that approximately 52% of caregivers of geriatric patients had depression scores that warranted further evaluation [25]. This aligns with our findings showing a prevalence of 45.4% with a 95% CI of 39.3 to 51.5%. Furthermore, depression is highlighted as one of the most common psychological consequences of caregiving, affecting caregivers of patients with severe mental illness and dementia [26]. Caregivers of individuals with chronic illnesses, such as dementia, cancer, or heart failure, are at increased risk of experiencing emotional distress and burnout [5,6,7]. The emotional impact of caregiving, combined with managing the care recipient’s health, can contribute to caregivers’ psychological strain and affect their quality of life [27].

The present study showed that the caregiving burden is an independent factor for depression, with bivariate analysis showing a positive relationship between the two scores. The analysis of the Zarit-12 scale score indicates that 43.5% of caregivers are likely to experience significant caregiver burden, i.e., moderate or high scores. Caregiver burden encompasses the physical, emotional, social, and financial stressors faced by individuals providing care to impaired or ill individuals, resulting in an imbalance between caregiving demands and personal resources [3]. Assessing this burden is relevant as it influences the potential need for patient institutionalization [8]. 

Consistent with our findings, research indicates a substantial impact of caregiving burden on mental health. Caregiver burden has been linked to adverse psychological outcomes, such as depression and anxiety [28,29]. Persistent burden predicts depressive symptoms in dementia caregivers, underscoring the long-term impact on mental health [30]. Additionally, caregiver burden correlates with symptoms of depression and anxiety and is associated with poorer physical and psychological health outcomes, including increased stress and reduced quality of life [31,32].

Additionally, caregiver burden can compromise caregivers’ overall well-being, impacting their ability to provide effective care [33]. Such effect extends across various caregiving contexts, such as caring for individuals with cancer, Parkinson’s disease, and other chronic conditions [34,35]. We observed that the most concerning aspects of caregiving among the participants were feelings of needing to do more and regret for not having taken better care of their relative. These perceptions may indicate a lack of personal resources or conflict between caregiving duties and the caregivers’ own needs. Additionally, given the relatively young age of the participants, such perceptions may relate to a lack of preparedness for caregiving or inadequate social support. Indeed, caregiver burden can interact with factors like preparedness for caregiving and social connectedness to impact caregivers’ health-related quality of life and psychological well-being [29,36]. Caregivers lacking support, resources, and coping mechanisms may experience elevated burden, leading to negative health outcomes for both the caregiver and the care recipient [37]. Seeking social support, building self-efficacy, and interventions focusing on enhancing emotional intelligence and resilience are beneficial for caregivers’ psychological well-being [27,38]. Further interventions proposed to alleviate caregiver burden include educational training programs and mindfulness interventions, aiming to reduce strain on caregivers and enhance their overall well-being [39,40]. 

The sum of this evidence indicates that caregiver burden is a multifaceted issue with significant implications for the health and well-being of caregivers and care recipients. Understanding the factors contributing to caregiver burden and implementing support strategies are crucial for promoting caregivers’ overall well-being and ensuring quality care for those in need of assistance.

### 4.3. Other Factors of Depression

Factors contributing to depression among caregivers of geriatric patients are also multidimensional and can be categorized into caregiver- and care-recipient-related factors. Among caregiver-related factors, younger age, female gender, single status, being a student, and having a low income were all associated with higher depression scores in the present study. However, only the female gender remained significant in the adjusted analysis. In line with these findings, female caregivers, lower education levels, and lower perceived social support have been linked to higher levels of caregiver depression [41]. These factors may have a differential impact depending on the care recipient’s condition. For example, caregivers of patients with dementia have been found to experience higher levels of depression, with female caregivers often exhibiting significantly higher depression scores compared to male caregivers [42]. The emphasis on the female gender is consistent with our findings, which show that female caregivers incur the highest risk of depression, as demonstrated in the adjusted analysis. On the other hand, social support was not investigated in the present study, although it constitutes a critical factor for a caregiver’s mental health. Research showed that social support and the caregiver’s perceived control over their lives can impact their risk of depression. Caregivers who experience social isolation, reduced control over their lives, and fear of inadequacy are at higher risk of depression [43]. This is likely consistent with our findings that show that sole caregivers and those living in the same household as the patient exhibit higher depression scores, although the analysis was not statistically significant. These findings highlight the importance of adequate social support and social connections in preventing depression among caregivers and mitigating eventual depressive symptoms. 

On the other hand, we observed a paradoxical finding where caregivers dedicating more than 8 h daily to caregiving experienced the lowest risk of depression compared to those who provided care less frequently, including those who did not provide care daily. This suggests that the caregiving burden in the studied population is likely influenced by other factors, possibly cultural and spiritual. Muslim caregivers often draw strength and guidance from their faith, incorporating Islamic principles into their caregiving practices. The doctrine of Allah and adherence to religious rituals provide comfort, resilience, and a sense of purpose, influencing their emotional well-being and coping strategies [44]. These beliefs and practices not only shape the caregivers’ approach to providing care but also influence their emotional well-being and coping strategies in the face of the challenges associated with caregiving. This dimension may explain why, in highly dedicated caregivers, the levels of depression are significantly lower, as the levels of dedication may correlate with higher spiritual value for caregiving. This relationship may relate to the dimension of preparedness for caregiving and social connectedness and their positive impact on caregivers’ psychological well-being [29,36]. 

Although not explored in the present study, patient-related factors, including the type and severity of the patient’s condition, cognitive impairment, and behavioral disturbances, also contribute to caregiver depression. Indeed, the burden of caregiving is more substantial when coupled with the challenges of caring for individuals with cognitive impairments, resulting in increased levels of depression among caregivers [45]. Caregivers of dementia patients, in particular, face challenges related to the care recipients’ cognitive impairments, which can heighten the burden and reduce caregivers’ well-being [40]. The severity of patients’ cognitive impairment is associated with increased levels of caregiver burden, emphasizing the relationship between patient condition and caregiver well-being [46]. The presence of chronic illness in elderly patients has also been identified as a risk factor for depression among caregivers [47]. Furthermore, caregivers of patients near the end of life, including those with dementia, have been found to experience high levels of anxiety and depression, underscoring the emotional toll of end-of-life care [48].

### 4.4. Implications for Action

The high prevalence of depression among caregivers of geriatric patients has significant public health implications, placing a considerable burden on healthcare resources and affecting the well-being of caregivers. Addressing this issue is crucial for improving the overall quality of care and societal well-being. Effective strategies should focus on providing mental health support, such as counseling and support groups, to alleviate the psychological strain on caregivers. Additionally, implementing respite care programs can offer temporary relief, reducing caregiver burnout. Respite care can take various forms, including in-home respite, short-term institutionalization, or adult day care, allowing caregivers a break from their caregiving responsibilities [49]. Studies have shown that respite care can effectively suppress caregiver burden, providing caregivers with much-needed relief from their caregiving duties [50,51]. Respite care allows caregivers to engage in self-care activities, reducing stress and improving their quality of life. Additionally, in the context of Saudi Arabia, such programs would be more acceptable culturally than complete institutionalization. 

Culturally sensitive interventions that build on Islamic values and practices can enhance the acceptability and effectiveness of these strategies within the community. Incorporating religious principles and rituals into caregiving support programs can provide caregivers with spiritual comfort, resilience, and a sense of purpose. Educational training programs tailored to the cultural context can further support caregivers in managing their burden [52,53]. 

Virtual education programs, such as the one evaluated by [54], have been effective in enhancing caregiver confidence and self-efficacy, demonstrating the potential of technology in delivering educational support. Moreover, the study by Parmar et al. (2022) emphasizes the importance of optimizing the integration of family caregivers in person-centered care through educational programs for the healthcare workforce [55].

By understanding and addressing the multifaceted nature of caregiver burden and the related factors and cultural dimensions, we can develop comprehensive support systems that promote caregivers’ mental health and well-being. These initiatives not only benefit caregivers but also improve the quality of care provided to geriatric patients, ultimately contributing to a healthier and more resilient society.

### 4.5. Limitations

This study has several limitations that should be acknowledged. First, the cross-sectional design limits the ability to establish causality between caregiver burden and depression. Longitudinal studies are needed to explore the temporal relationship between these variables. Second, the use of self-reported questionnaires for assessing depression and caregiver burden may introduce response bias, as participants might underreport or overreport their symptoms and experiences. Third, the snowball sampling technique might have led to selection bias, as the initial participants and their networks may not be representative of the broader population of caregivers. Fourth, the study was conducted at a single hospital in Jeddah, Saudi Arabia, which may limit the generalizability of the findings to other regions and healthcare settings. Additionally, cultural factors specific to Saudi Arabia may influence caregiving experiences and the prevalence of depression, making it difficult to generalize the results to other cultural contexts. Another limitation is the retrospective inclusion of younger caregivers (aged 12–17) despite the initial inclusion criteria specifying participants aged 18 and older. While this decision enriches the cultural context of the findings, it may introduce concerns regarding the validity of the tools used for this younger population. However, given that these participants represent less than 4% of the sample, the overall validity remains largely unaffected. Lastly, the study did not consider care recipients’ demographic and clinical factors, which have valuable implications for caregiver burden and the risk of depression.

## 5. Conclusions

This study highlights the significant prevalence of depression among caregivers of geriatric patients in Jeddah, Saudi Arabia, and emphasizes the crucial impact of caregiving burden on mental health. Our findings showed that younger age, female gender, single status, being a student, and low income were associated with higher depression scores among caregivers. Caregiving burden, as measured by the ZBI-12, was a strong predictor of depression, underscoring the need for effective support systems for caregivers.

Addressing these challenges aligns with the goals of Saudi Vision 2030, which emphasizes longevity and healthiness among the population. Targeted interventions such as mental health support, respite care programs, and culturally sensitive educational training are essential to mitigate caregiver burden and improve mental health outcomes. These strategies can help enhance the overall quality of care provided to geriatric patients, contributing to a more efficient healthcare system and reducing economic burdens.

By developing comprehensive support systems that consider the unique needs and cultural contexts of caregivers, we can promote their well-being and ensure better outcomes for both caregivers and the geriatric population they serve. Future research should focus on longitudinal studies to better understand the causal relationships and explore the effectiveness of various interventions in reducing caregiver burden and associated depression. This approach will not only support caregivers but also contribute significantly to the broader goals of Saudi Vision 2030, fostering a healthier, more resilient society.

## Figures and Tables

**Table 1:** Participant characteristics (n = 269).

Parameter	Level	Mean	SD
Age	(years)	31.96	15.01
	Range	12	77
Parameter	Level	Frequency	Percentage
Gender	Male	132	49.1
	Female	137	50.9
Marital status	Single	171	63.6
	Married	80	29.7
	Divorced	13	4.8
	Widowed	5	1.9
Education level	Uneducated	3	1.1
	Secondary school	57	21.2
	College (not graduated)	82	30.5
	Bachelor’s	104	38.7
	Post-graduate	23	8.6
Professional status	Employed	81	30.1
	Student	113	42.0
	Retired	30	11.2
	Unemployed	45	16.7
Monthly income	<3K SAR	89	33.1
	3K–6K SAR	51	19.0
	6K–10K SAR	53	19.7
	>10K SAR	76	28.3

**Table 2:** Caregiving parameters.

Parameter	Level	Frequency	Percentage
Relationship to the patient	Son/Daughter	133	49.4
	Brother/Sister	43	16.0
	Spouse	10	3.7
	Father/Mother	4	1.5
	Grandparent	23	8.6
	Grandchild	20	7.4
	Other	30	11.2
	Not mentioned	6	2.2
Caregiving frequency	>8 h daily	59	21.9
	<8 h daily	104	38.7
	Not daily	106	39.4
Same habitat with the patient	No	111	41.3
	Yes	158	58.7
Caregiving mode	In alternance	219	81.4
	Sole caregiver	50	18.6
Financial responsibility	No	200	74.3
	Yes	69	25.7
Caregiving duration	Less than 6 months	94	34.9
	More than 6 months	175	65.1

**Table 3:** Answers to the Patient Health Questionnaire-9 Items (PHQ-9) scale.

Item	Never	A Few Times	Sometimes	Always	Mean (SD)
Losing interest or pleasure in doing things	86 (32.0)	89 (33.1)	77 (28.6)	17 (6.3)	2.09 (0.92)
Feeling sad or unhappy	107 (39.8)	73 (27.1)	72 (26.8)	17 (6.3)	2.00 (0.96)
Difficulty sleeping or staying asleep	91 (33.8)	54 (20.1)	87 (32.3)	37 (13.8)	2.26 (1.07)
Feeling tired or having little energy	70 (26.0)	72 (26.8)	87 (32.3)	40 (14.9)	2.36 (1.03)
Poor appetite or overeating	115 (42.8)	65 (24.2)	62 (23.0)	27 (10.0)	2.00 (1.03)
Feeling bad about yourself or that you are a failure	143 (53.2)	59 (21.9)	41 (15.2)	26 (9.7)	1.81 (1.02)
Trouble concentrating	102 (37.9)	84 (31.2)	59 (21.9)	24 (8.9)	2.02 (0.98)
Moving or speaking so slowly or the opposite–being so fidgety or restless	151 (56.1)	54 (20.1)	50 (18.6)	14 (5.2)	1.73 (0.94)
Feeling as though life isn’t worth living	170 (63.2)	41 (15.2)	38 (14.1)	20 (7.4)	1.66 (0.98)

Cronbach’s alpha = 0.890.

**Table 4:** Answers to the Short Form Zarit Burden Interview (ZBI-12) scale.

Item	Never	Rarely	Sometimes	Often	Almost Always	Mean (SD)
Do you feel that because of the time you spend with your relative, you do not have enough time for yourself?	117 (43.5)	57 (21.2)	70 (26.0)	21 (7.8)	4 (1.5)	2.03 (1.07)
Do you feel conflicted and stressed between worrying about your relative and other duties such as your job or family?	111 (41.3)	59 (21.9)	64 (23.8)	27 (10.0)	8 (3.0)	2.12 (1.15)
Do you feel angry when you are near your relative?	154 (57.2)	50 (18.6)	46 (17.1)	13 (4.8)	6 (2.2)	1.76 (1.04)
Do you feel that your relative negatively affects your relationships with other family members?	154 (57.2)	44 (16.4)	41 (15.2)	27 (10.0)	3 (1.1)	1.81 (1.09)
Do you feel tense towards your relative?	149 (55.4)	48 (17.8)	47 (17.5)	21 (7.8)	4 (1.5)	1.82 (1.07)
Do you feel that you do not have enough privacy because of your relative?	154 (57.2)	55 (20.4)	35 (13.0)	22 (8.2)	3 (1.1)	1.75 (1.04)
Do you feel that your health has been affected after taking part in caring for your relative?	172 (63.9)	38 (14.1)	43 (16.0)	12 (4.5)	4 (1.5)	1.65 (1.00)
Do you feel that your social life has been harmed because of your relative?	148 (55.0)	46 (17.1)	42 (15.6)	23 (8.6)	10 (3.7)	1.89 (1.17)
Do you feel that you have lost control of your life since your relative became ill?	159 (59.1)	49 (18.2)	38 (14.1)	17 (6.3)	6 (2.2)	1.74 (1.06)
Do you feel unsure about the proper treatment for your relative?	142 (52.8)	53 (19.7)	47 (17.5)	24 (8.9)	3 (1.1)	1.86 (1.07)
Do you feel that you need to do more for your relative?	85 (31.6)	59 (21.9)	63 (23.4)	31 (11.5)	31 (11.5)	2.49 (1.35)
Do you feel that you could have taken better care of your relative?	89 (33.1)	52 (19.3)	59 (21.9)	45 (16.7)	24 (8.9)	2.49 (1.34)

Values are frequencies (percentages); Cronbach’s alpha = 0.912.

**Table 5:** Factors associated with the likelihood of depression indicated by PHQ-p score ≥ 10.

Factor	Level	Depression Status				*p*-Value
		No (PHQ-9 Score < 10)		Yes (PHQ-9 Score > = 10)		
Age	(mean, SD)	35.37	15.33	27.84	13.58	<0.001 *^§^
Gender	Male	87	65.9	45	34.1	
	Female	60	43.8	77	56.2	<0.001 *
Marital status	Single	76	44.4	95	55.6	
	Married	59	73.8	21	26.3	
	Divorced	9	69.2	4	30.8	
	Widowed	3	60.0	2	40.0	<0.001 *
Education level	Uneducated	2	66.7	1	33.3	
	Secondary	30	52.6	27	47.4	
	College	42	51.2	40	48.8	
	Bachelor’s	55	52.9	49	47.1	
	Post-graduate	18	78.3	5	21.7	0.202
Professional status	Employed	53	65.4	28	34.6	
	Student	46	40.7	67	59.3	
	Retired	22	73.3	8	26.7	
	Unemployed	26	57.8	19	42.2	0.001 *
Monthly income	<3K SAR	39	43.8	50	56.2	
	3K–6K SAR	22	43.1	29	56.9	
	6K–10K SAR	31	58.5	22	41.5	
	>10K SAR	55	72.4	21	27.6	0.001 *
Relationship to the patient	Son/Daughter	80	60.2	53	39.8	
	Brother/Sister	16	37.2	27	62.8	
	Spouse	4	40.0	6	60.0	
	Father/Mother	3	75.0	1	25.0	
	Grandparent	15	65.2	8	34.8	
	Grandchild	10	50.0	10	50.0	
	Other	17	56.7	13	43.3	
	Not mentioned	2	33.3	4	66.7	0.148
Caregiving frequency	>8 h daily	40	67.8	19	32.2	
	<8 h daily	48	46.2	56	53.8	
	Not daily	59	55.7	47	44.3	0.028 *
Same habitat with the patient	No	68	61.3	43	38.7	
	Yes	79	50.0	79	50.0	0.068
Caregiving mode	In alternance	123	56.2	96	43.8	
	Sole caregiver	24	48.0	26	52.0	0.295
Financial responsibility	No	105	52.5	95	47.5	
	Yes	42	60.9	27	39.1	0.229
Caregiving duration	<6 months	50	53.2	44	46.8	
	>6 months	97	55.4	78	44.6	0.725
Caregiving burden (ZBI-12 score)	None-to-mild (0–10)	114	75.0	38	25.0	
	Mild-to-moderate (10–20)	24	39.3	37	60.7	
	High (>20)	9	16.1	47	83.9	<0.001 *

Values are frequencies (percentages). Test used: ^§^ independent t-test; otherwise, the chi-square test was used. * Statistically significant result (*p* < 0.05).

**Table 6:** Independent factors of depression indicated by PHQ-p score ≥ 10 (multivariate regression model—Enter method).

Predictor	Level	OR	95%CI		*p*-Value
Age	(mean, SD)	0.97	0.94	1.00	0.089
Gender	Female	2.50	1.30	4.80	0.006 *
Marital status	Single ^§^	Ref	-	-	0.314
	Married	0.39	0.14	1.10	0.075
	Divorced	0.47	0.08	2.76	0.406
	Widowed ^§^	1.50	0.15	15.17	0.730
Professional status	Employed	Ref	-	-	0.794
	Student ^§^	1.35	0.51	3.55	0.541
	Retired	1.76	0.49	6.26	0.386
	Unemployed ^§^	1.19	0.42	3.33	0.742
Monthly income	<3K SAR	0.97	0.39	2.39	0.945
	3K–6K SAR	2.43	0.95	6.23	0.064
	6K–10K SAR	1.37	0.53	3.53	0.518
	>10K SAR	Ref	-	-	0.194
Caregiving frequency	>8 h daily	Ref	-	-	0.006 *
	<8 h daily	4.21	1.69	10.49	0.002 *
	Not daily	3.76	1.51	9.35	0.004 *
Caregiving burden (ZBI-12 score)	None-to-mild (0–10)	Ref	-	-	<0.001 *
	Mild-to-moderate (10–20)	6.18	2.94	13.00	<0.001 *
	High (>20)	22.75	8.75	59.13	<0.001 *

^§^ These categories were analyzed as dummy variables in another model but showed no significance. The overall model explained 46.4% of the variance of the outcome variable (R^2^ = 0.464). * Statistically significant difference (*p* < 0.05).

## Data Availability

Data for this study are available upon reasonable request from the first author.
